# Synthesis, Characterization, Anticancer, and Antioxidant Studies of Ru(III) Complexes of Monobasic Tridentate Schiff Bases

**DOI:** 10.1155/2016/9672451

**Published:** 2016-08-11

**Authors:** Ikechukwu P. Ejidike, Peter A. Ajibade

**Affiliations:** Department of Chemistry, Faculty of Science and Agriculture, University of Fort Hare, P.B. X1314, Alice 5700, South Africa

## Abstract

Mononuclear Ru(III) complexes of the type [Ru(LL)Cl_2_(H_2_O)] (LL = monobasic tridentate Schiff base anion: (1*Z*)-*N*′-(2-{(*E*)-[1-(2,4-dihydroxyphenyl)ethylidene]amino}ethyl)-*N*-phenylethanimidamide [DAE], 4-[(1*E*)-*N*-{2-[(*Z*)-(4-hydroxy-3-methoxybenzylidene)amino]ethyl}ethanimidoyl]benzene-1,3-diol [HME], 4-[(1*E*)-*N*-{2-[(*Z*)-(3,4-dimethoxybenzylidene)amino]ethyl}ethanimidoyl]benzene-1,3-diol [MBE], and* N*-(2-{(*E*)-[1-(2,4-dihydroxyphenyl)ethylidene]amino}ethyl)benzenecarboximidoyl chloride [DEE]) were synthesized and characterized using the microanalytical, conductivity measurements, electronic spectra, and FTIR spectroscopy. IR spectral studies confirmed that the ligands act as tridentate chelate coordinating the metal ion through the azomethine nitrogen and phenolic oxygen atom. An octahedral geometry has been proposed for all Ru(III)-Schiff base complexes.* In vitro* anticancer studies of the synthesized complexes against renal cancer cells (TK-10), melanoma cancer cells (UACC-62), and breast cancer cells (MCF-7) was investigated using the Sulforhodamine B assay. [Ru(DAE)Cl_2_(H_2_O)] showed the highest activity with IC_50_ valves of 3.57 ± 1.09, 6.44 ± 0.38, and 9.06 ± 1.18 *μ*M against MCF-7, UACC-62, and TK-10, respectively, order of activity being TK-10 < UACC-62 < MCF-7. The antioxidant activity by DPPH and ABTS inhibition assay was also examined. Scavenging ability of the complexes on DPPH radical can be ranked in the following order: [Ru(DEE)Cl_2_(H_2_O)] > [Ru(HME)Cl_2_(H_2_O)] > [Ru(DAE)Cl_2_(H_2_O)] > [Ru(MBE)Cl_2_(H_2_O)].

## 1. Introduction

Coordination chemistry of transition metal Schiff base complexes possessing N, O, and S-donor atoms has received consideration over the past few decades, due to the imperative roles these compounds have played in a variety of biochemical procedures like haloperoxidation [1], insulin mimicking [2, 3], fixation of nitrogen [4], inhibition of cancer growth, and prophylaxis against carcinogenesis [5, 6]. A huge variety of carbonyl compounds (>C=O) and amines (R-NH_2_) have been exploited in the preparation of Schiff bases [7, 8]. The reactivity of aldehyde compounds is generally faster than those of the ketones in condensation reaction, thereby resulting in the formation of Schiff bases with a centre that are less steric than the ketone's, relatively unstable and freely polymerizable [9]. This important attribute of Schiff base ligands offers prospects for prompting substrate chirality and metal centred electronic factor tuning and improving the solubility and steadiness of either homogeneous or heterogeneous catalysts [10–12].

Schiff bases have shown an interesting application as an active corrosion inhibitor that is established on their capability to spontaneously form a monolayer upon the surface to be glazed [13], as it is a type of interaction existing between an inhibitor and a metal surface known as chemisorption [14]. It is interesting to note that several commercial inhibitors contain amines and aldehydes, but seemingly because of the presence of >C=N bond, this makes Schiff bases function more resourcefully in many ways [15]. Stabilization of metal ions in various oxidation states and monitoring their reactivity for catalytic applications have been linked to Schiff bases [16]. The nitrogen-oxygen Schiff bases geometry largely relies on the diamine structural unit, nature of the ancillary ligand, and the central metal ion [17]. Schiff base-transition metal complexes have been known to be one of the most modifiable and comprehensively studied systems [18] with applications in clinical and analytical fields [19, 20]. Antioxidants derived from metal Schiff base ligand combinations have received current attention for their capability to safeguard living systems and cells from impairment caused by oxidative stress or free radicals [21].

DNA binding, cleavage potentials, scavenging potentials, and anticancer investigations of Schiff base-ruthenium(III) complexes have been accounted for [22]. Synthesis, spectral, redox, catalytic, and biological action investigation of mononuclear Ru(III)-Schiff base structures are reported [23]. 2,2′-Bipyridine and tetradentate Schiff base ancillary ligands of mixed-ligand Ru(II) complexes have been reported for their electrochemical and Na^+^ binding properties [24]. Catalytic and growth inhibitory activities of Ru(III) mixed ligand complexes of 2-hydroxy-1-naphthylideneimines have been reported [25].

**Scheme 1:**
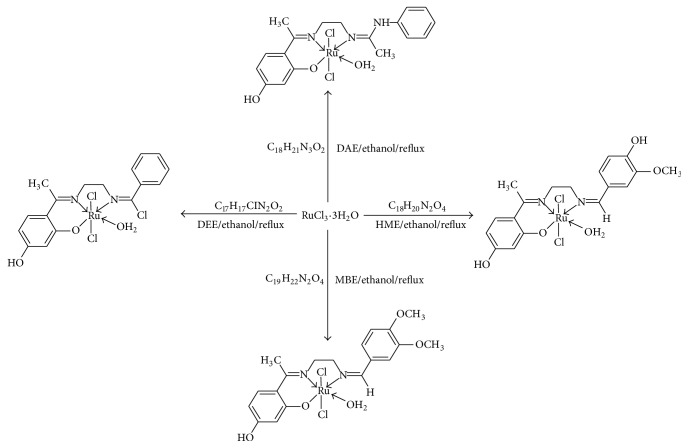
Structure of mononuclear ruthenium(III)-Schiff base complexes.

In this study, we report the synthesis, characterization, free radical scavenging, and anticancer studies of four mononuclear ruthenium(III) complexes of Schiff bases derived from 2′,4′-dihydroxyacetophenone and ethylenediamine as the bridging ligand with RCHO moiety alongside their radicals scavenging action on 1,1-diphenyl-2-picrylhydrazyl (DPPH) and 2,2′-azino-bis(3-ethylbenzothiazoline-6-sulfonic acid) (ABTS) and antiproliferative potentials. The Schiff base ligands containing N_2_O type tridentate partitions were utilized for the synthesis of the mononuclear ruthenium(III)-Schiff base complexes (Scheme 1).

## 2. Experimental

### 2.1. Chemicals and Instrumentations

All reagents used were of analytical grade and used as purchased commercially. Ethylenediamine,* N,N*′-dimethylformamide (DMF) and ascorbic acid (Vit. C) were received from Merck, 2′,4′-dihydroxyacetophenone and RuCl_3_·3H_2_O were obtained from Aldrich. 1,1-Diphenyl-2-picrylhydrazyl (DPPH), 2,2′-azinobis-3-ethylbenzothiazoline-6-sulfonic acid (ABTS), butylated hydroxytoluene (BHT), and rutin hydrate were received from Sigma Chemical Co. (St. Louis, MO, USA). Elemental analysis was carried out using Perkin-Elmer elemental analyzer. IR spectra were recorded on an FT-IR spectrometer: Perkin-Elmer System (Spectrum 2000) via KBr disk method was used for the IR spectra analysis. Freshly prepared DMF solutions of about 10^−3 ^M containing Ru(III) complexes gave the molar conductance at room temperature with Crison EC-Meter Basic 30+ conductivity cell. Electronic absorption spectra ranging from 200 to 900 nm were recorded on a Perkin-Elmer Lambda-25 spectrophotometer. Stuart melting point (SMP 11) was used for the melting points. Four N_2_O type tridentate ligands, (1*Z*)-*N*′-(2-{(*E*)-[1-(2,4-dihydroxyphenyl)ethylidene]amino}ethyl)-*N*-phenylethanimidamide [DAE], 4-[(1*E*)-*N*-{2-[(*Z*)-(4-hydroxy-3-methoxybenzylidene)amino]ethyl}ethanimidoyl]benzene-1,3-diol [HME], 4-[(1*E*)-*N*-{2-[(*Z*)-(3,4-dimethoxybenzylidene)amino]ethyl}ethanimidoyl]benzene-1,3-diol [MBE], and* N*-(2-{(*E*)-[1-(2,4-dihydroxyphenyl)ethylidene]amino}ethyl)benzenecarboximidoyl chloride [DEE], were synthesized and reported previously [26].

### 2.2. Preparation of the Tridentate Schiff Bases (DAE, HME, MBE, and DEE)

Ethylenediamine (0.015 mol) dissolved in 20 mL of alcohol was slowly added to 2′,4′-dihydroxyacetophenone (0.015 mol) dissolved in same alcohol (30 mL) and allowed to stir for 60 minutes at room temperature and then followed by drop-wise addition of appropriate aldehyde (RCHO, 15 mmol) dissolved in 30 mL alcohol for 20 minutes time interval at room temperature and further stirred for 120 minutes. The mixture was left standing with continuous stirring for approximately 36 hours at room temperature, after which the desired tridentate compounds were filtered and washed with ethanol to give crystalline solid. The crude product was recrystallized from warm ethanol. The products were dried in the vacuum at 50°C overnight to give analytically pure products in good yields (64.2% to 73.8%).

### 2.3. Synthesis of Ru(III)-Tridentate Schiff Base Complexes

Ru(III) complexes were prepared by adding (0.5 mmol) ethanol solution of ruthenium(III) chloride to a warm ethanolic solution (0.5 mmol) of [DAE]/[HME]/[MBE]/[DEE], respectively. The colour of the solutions changed immediately, magnetically stirred and kept under reflux for 6 hours. The precipitated solids were filtered by suction from the reaction medium, washed with ethanol and then with diethyl ether, and dried over anhydrous calcium chloride. The yields were about 55.7–61.9%. The synthesis of the complexes is explained in Scheme 1.

#### 2.3.1. Synthesis of [OHC_6_H_3_OH:C(CH_3_):N(C_2_H_4_)N:C(CH_3_):NHC_6_H_5_RuCl_2_(H_2_O)]

*[Ru(DAE)Cl*_*2*_ *(H*_*2*_ *O)]·H*_*2*_ *O.* Dark-green solid; Yield: 156.6 mg (60.4%); F. Wt: 518.38 g; Anal. Calcd. for C_18_H_24_N_3_O_4_RuCl_2_ (%): C 41.71, H 4.67, N 8.11; Found (%): C 41.43, H 4.54, N 8.29; IR (KBr) *ν*_max_/cm^−1^: 3436 (O-H), 1621 (C=N), 1242, 1170 (C-O), 520 (Ru-N), 438 (Ru-O); UV-Vis (DMF): *λ*_max_/nm (cm^−1^): 281 (35 587), 310 (32 258), 391 (25 576), 452 (22 124), 525 (19 048), 613 (16 313); Decomp. Temp, °C, 238-239°C; Λ_*μ*_: 31.8 *μ*Scm^−1^.

#### 2.3.2. Synthesis of [OHC_6_H_3_OH:C(CH_3_):N(C_2_H_4_)N:CH:C_6_H_3_OHOCH_3_RuCl_2_(H_2_O)]

*[Ru(HME)Cl*_*2*_ *(H*_*2*_ *O)]·H*_*2*_ *O.* Darkish-green Solid; Yield: 165.7 mg (61.9%); F. Wt: 535.37 g; Anal. Calcd. for C_18_H_23_N_2_O_6_RuCl_2_ (%): C 40.38, H 4.33, N 5.23; Found (%): C 40.58, H 4.21, N 5.44; IR (KBr) *ν*_max_/cm^−1^: 3422 (O-H), 1637 (C=N), 1245, 1173 (C-O), 485 (Ru-N), 437 (Ru-O); UV-Vis (DMF): *λ*_max_/nm (cm^−1^): 277 (36 101), 309 (32 363), 381 (26 247), 393 (25 446), 513 (19 493), 623 (16 051); Decomp. Temp, °C, 218-219°C; Λ_*μ*_: 30.5 *μ*Scm^−1^.

#### 2.3.3. Synthesis of [OHC_6_H_3_OH:C(CH_3_):N(C_2_H_4_)N:CH:C_6_H_5_(OCH_3_)_2_RuCl_2_(H_2_O)]

*[Ru(MBE)Cl*_*2*_ *(H*_*2*_ *O)]·H*_*2*_ *O.* Darkish-green Solid; Yield: 160.4 mg (58.4%); F. Wt: 549.39 g; Anal. Calcd. for C_19_H_25_N_2_O_6_RuCl_2_ (%): C 41.54, H 4.59, N 5.10; Found (%): C 41.29, H 4.32, N 4.98; IR (KBr) *ν*_max_/cm^−1^: 3435 (O-H), 1639 (C=N), 1244, 1171 (C-O), 548 (Ru-N), 475 (Ru-O); UV-Vis (DMF): *λ*_max_/nm (cm^−1^): 277 (36 101), 311 (32 155), 380 (26 316), 393 (25 446), 510 (19 608), 623 (16 051); Decomp. Temp, °C, 226-227°C; Λ_*μ*_: 30.1 *μ*Scm^−1^.

#### 2.3.4. Synthesis of [OHC_6_H_3_OH:C(CH_3_):N(C_2_H_4_)N:C(Cl):C_6_H_5_RuCl_2_(H_2_O)]

*[Ru(DEE)Cl*_*2*_ *(H*_*2*_ *O)]·H*_*2*_ *O.* Dark-green Solid; Yield: 145.9 mg (55.7%); F. Wt: 523.79 g; Anal. Calcd. for C_17_H_20_N_2_O_4_RuCl_3_ (%): C 38.98, H 3.85, N 5.35; Found (%): C 39.11, H 3.67, N 5.11; IR (KBr) *ν*_max_/cm^−1^: 3416 (O-H), 1617 (C=N), 1243, 1169 (C-O), 475 (Ru-N), 436 (Ru-O); UV-Vis (DMF): *λ*_max_/nm (cm^−1^): 275 (31 364), 306 (32 680), 385 (25 974), 521 (19 231), 632 (15 823); Decomp. Temp, °C, 228-229°C; Λ_*μ*_: 38.8 *μ*Scm^−1^.

### 2.4. *In Vitro *Antiproliferative Activity

The potentials of the Ru(III)-tridentate Schiff base complexes to interfere with the growth of TK-10 renal cell line, UACC-62 melanoma cell line, and MCF-7 breast cell lines were determined by SRB assay as previously described [22]. 3–19 passages of MCF-7, TK-10, and UACC-62 cell lines with plating densities of 7–10 000 cells per well were precultured into 96-well microtitre plates for 24 h at 37°C with 95% air, 5% CO_2_, and 100% relative humidity in RPMI medium, supplemented with 5% fetal bovine serum (FBS), 50 *μ*g mL^−1^ (gentamicin), and 2 mM L-glutamine [27]. The compounds were dissolved in DMSO and treated with the cells after 24 h and diluted in RPMI medium giving rise to 5 concentrations comprising 0.01, 0.1, 0, 10, and 100 *μ*M.

Wells containing culture medium were used as control while the wells containing complete culture medium with no cells were used as the blanks. Parthenolide was used as the standard drug in this study. The plates were then incubated for 48 h after the addition of the compounds. Viable cells were fixed to the bottom of each well with cold 50% trichloroacetic acid, washed, dried, and dyed by SRB. The unbounded dye was separated, while the protein-bound dye was extracted with 10 mM Tris base and multiwell spectrophotometer at the wavelength 540 nm was used for its optical density determination. IC_50_ values were determined by plotting the percentage viability against concentration of compounds on a logarithmic graph to obtain 50% of cell growth inhibition relative to the control.

### 2.5. Antioxidant Assay

#### 2.5.1. Scavenging Activity of 1,1-Diphenyl-2-picrylhydrazyl (DPPH) Radical

The antioxidant activity of the prepared Ru(III) complexes was studied using spectrophotometer by 1,1-diphenyl-2-picrylhydrazyl (DPPH) method. This compound is known as a stable readily accessible free radical, with solubility in methanol giving a purple solution, and when reacted with antioxidant species changes to an equivalent light yellow colour. The radical scavenging potentials of the complexes with DPPH radical were evaluated as described [22]. 1 mL solution of the compounds in DMF with concentrations ranging from 100 to 500 *μ*g/mL was mixed thoroughly with equivalent amount of 0.4 mM DPPH in methanol; the mixtures were then allowed to react in the dark for half an hour. Measurement of the mixture absorbance was achieved spectrophotometrically at 517 nm. Vitamin C and rutin were used as the standard drugs. All test analysis was carried out in triplicate. The ability of the ruthenium compounds to scavenge DPPH radical was calculated via the following equation:

(1) DPPH  radical  scavenging  activity  %=Absorbance  of  control−Absorbance  of  sampleAbsorbance  of  control×100.

#### 2.5.2. ABTS: 2,2′-Azino-bis(3-ethylbenzothiazoline-6-sulfonic acid) Radical Scavenging Assay

ABTS scavenging ability of the Ru(III)-tridentate Schiff base complexes adopted a described method [28]. 7 mM ABTS solution and 2.4 mM potassium persulfate solution in equal amounts (1 : 1) were used for working solution preparation and allowed to react in the dark for 12 h at room temperature. An absorbance of 0.706 ± 0.001 units at 734 nm required for the analysis was obtained by diluting 1 mL ABTS^+^ solution. Test samples (1 mL) were mixed with 1 mL of the ABTS^+^ solution, and absorbance was read spectrophotometrically at 734 nm. The test samples' ABTS scavenging capacity alongside standard drugs was evaluated. Triplicate analysis was carried out. The percentage inhibition of ABTS radical scavenging activity was obtained following a previous report [28].

## 3. Results and Discussion

### 3.1. Synthesis and Characterization

The obtained compounds were of coloured powders, stable in atmosphere with a general formula: [Ru(LL)Cl_2_(H_2_O)] (LL = monobasic tridentate Schiff base anion: DAE, HME, MBE, and DEE). They were prepared by treating [RuCl_3_·3H_2_O] with the corresponding Schiff base in an equal mole ratio in alcohol as depicted in the Scheme 1. All the complexes are dark-green and sparingly soluble in general organic solvents but soluble in polar aprotic solvent such as DMF and DMSO; the melting point analysis showed that the Ru(III) complexes were decomposing before melting. The physicoanalytical data collected for the compounds are in agreement with the structural formulae proposed, thus confirming the suggested mononuclear composition for the Ru(III) complexes (Scheme 1).

### 3.2. Molar Conductivity Measurements

The molar conductance of the synthesized Ru(III) complexes was measured in DMF at 10^−3 ^M solution. The values were found to be in the range of 30.1–38.8 *μ*Scm^−1^ suggesting the nonelectrolytic nature of the complexes in solution [22, 29].

### 3.3. Infrared Spectra

Valuable evidence concerning the environment of the functional group attached to the ruthenium atom has been obtained from the FTIR spectra. The IR spectra of the ligands, when compared with those of the newly synthesized complexes, confirm the coordination of N_2_O type tridentate ligands to the ruthenium ion. The classification was achieved by comparing the spectra of the ligands with those originating from the coordination between ruthenium(III) metal ion and the active sites. The Schiff bases showed the broad bands in the 3462–3477 cm^−1^ range attributable to the *ν*(OH) cm^−1^ vibration. Ligand infrared spectra showed that a band at 1605–1619 cm^−1^ is attributed to *ν*(C=N) stretching of the azomethine group based on earlier reports [30]. This *ν*(C=N) shift to 1617–1639 cm^−1^ in all the complexes by about 5–23 cm^−1^ signifies the participation of azomethine nitrogen in the coordination sphere with the ruthenium(III) ion for all the complexes [21, 31]. A medium band that corresponds to phenolic oxygen atom *ν*(C-O) is observed at 1167 and 1245 cm^−1^ for the free ligands.

The higher shifting of *ν*(C-O) stretching vibrations as observed in the ruthenium(III) complexes spectra suggests that the phenolic OH group of Schiff base, DAE, HME, MBE, and DEE, is involved in coordination with ruthenium ion after deprotonation [32, 33]. Seemingly, the DAE, HME, MBE, and DEE ligands act as a tridentate chelating compound, coordinating to the metal ion via the two nitrogen atoms of the azomethine group as well as O atom of phenolic group [21, 25]. This is further supported by the displacement of *ν*(O-H) in the range 3462–3477 cm^−1^ in all the complexes. The presence of coordinated water gave a broad band that appeared in the regions 3416–3436 and 813–851 cm^−1^; this can be due to *ν*(O-H) stretching and *ν*(O-H) rocking vibrations, respectively, which further confirms the presence of nonligand assignable to the rocking mode of water [28, 34]. New weak nonligand bands that are not found in the DAE, HME, MBE, and DEE ligands appeared in the ranges 475–548 cm^−1^ and 436–475 cm^−1^ in the complexes spectra attributed to *ν*(Ru-N) and *ν*(Ru-O) vibrations, respectively [35, 36]. A band ranging from 311–346 cm^−1^ appeared in the spectra of the Ru(III)-Schiff base complexes indicating the presence of two chloride ions in* trans* position around ruthenium centre [37–40].

### 3.4. Electronic Absorption Spectra Studies

**Figure 1:**
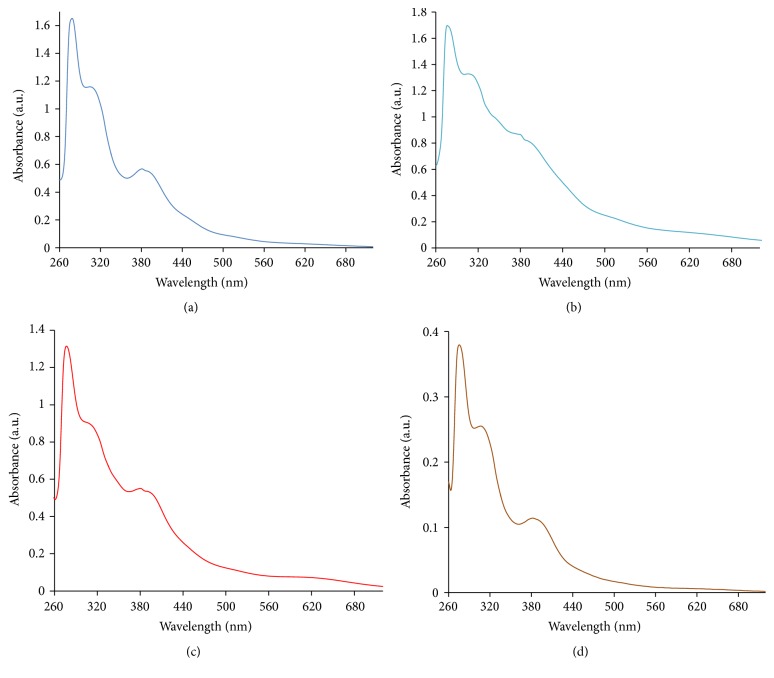
Electronic absorption spectra of the Ru(III) complexes: (a) [Ru(DAE)Cl_2_(H_2_O)]; (b) [Ru(HME)Cl_2_(H_2_O)]; (c) [Ru(MBE)Cl_2_(H_2_O)]; (d) [Ru(DEE)Cl_2_(H_2_O)].

The UV-Vis spectra of the Ru(III)-Schiff base complexes in DMF solutions were recorded at room temperature ranging from 200 to 900 nm. The nature of DAE, HME, MBE, and DEE ligands field around the ruthenium ion was obtained from the electronic spectra. The free ligands showed absorption bands within the range of 277–393 nm attributable to *π* ^*∗*^ ← *π* and *π* ^*∗*^ ← *n* transitions relating the benzene ring (Figure 1). The shifting of these bands in the complexes spectra followed the participation of the imine group nitrogen and phenolic group oxygen in bonding [22, 25]. Ground state of ruthenium(III) is ^2^T_2g_, where initial excited doublet levels in order of increasing energy are ^2^A_2g_ and ^2^T_1g_, arising from t_2g_^4^e_g_^1^ configuration [41].

Ru^3+^ ion, with a d^5^ electronic configuration, possesses high oxidizing properties and large crystal field parameter. Also, charge transfer bands of the type L_*π*y_ → T_2g_ were noticeable within low energy region, obscuring weaker bands that is due to d-d transitions [22, 25]. The extinction coefficient bands around 613–632 nm regions are found to be low when compared to the charge transfer bands. These bands have been assigned to ^2^T_2g_ → ^2^A_2g_ transition and are in agreement with the assignment made for similar octahedral ruthenium(III) complexes [42, 43]. Absorption bands within the 452–525 nm regions were assigned to the charge transfer transitions [22, 44]. Overall, the absorption spectra of the Ru(III)-Schiff base complexes are typical of octahedral environment about the ruthenium(III) ions [22].

### 3.5. Antiproliferative Activity

Investigation into the structure-activity relationship of the isolated Ru(III)-N_2_O Schiff base complexes with respect to different functional groups on the ligands used for ruthenium ion complex formation has been conducted via antiproliferative studies. Three of the Ru(III)-Schiff base compounds alongside parthenolide were subjected to cell lines tests at different sample concentrations ranging from 0.01 to 100 *μ*M towards renal cancer cell (TK-10), melanoma cancer cell (UACC-62), and breast cancer cell (MCF-7). The cancer cell lines were incubated for 48 h, followed by the addition of the compounds of various concentrations via Sulforhodamine B (SRB) assay [22].

**Figure 2:**
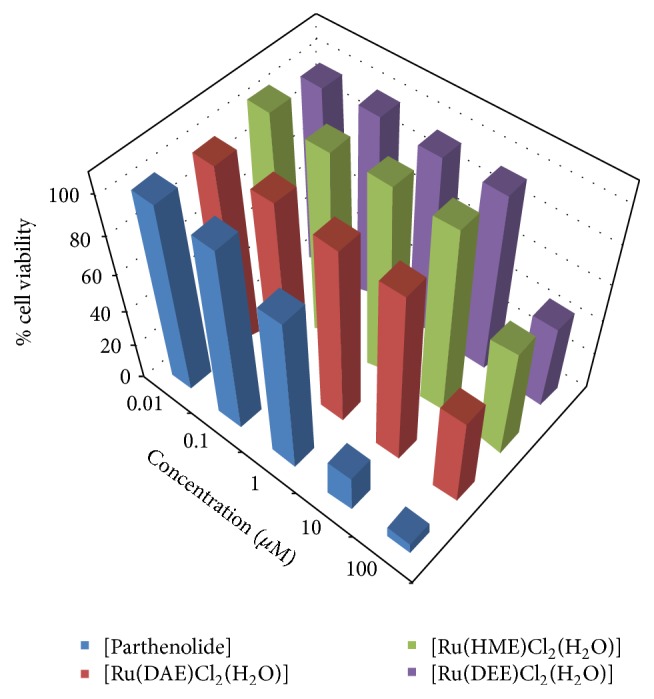
*In vitro* antiproliferative activity of Ru(III) complexes and parthenolide against human breast cancer cell line (MCF-7).

**Figure 3 fig3:**
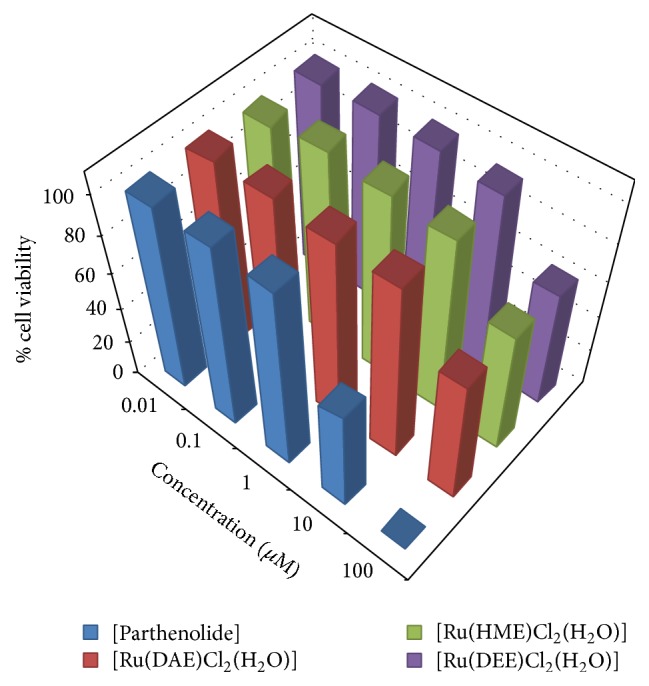
*In vitro* antiproliferative activity of Ru(III) complexes and parthenolide against human melanoma cancer cell (UACC-62).

**Figure 4:**
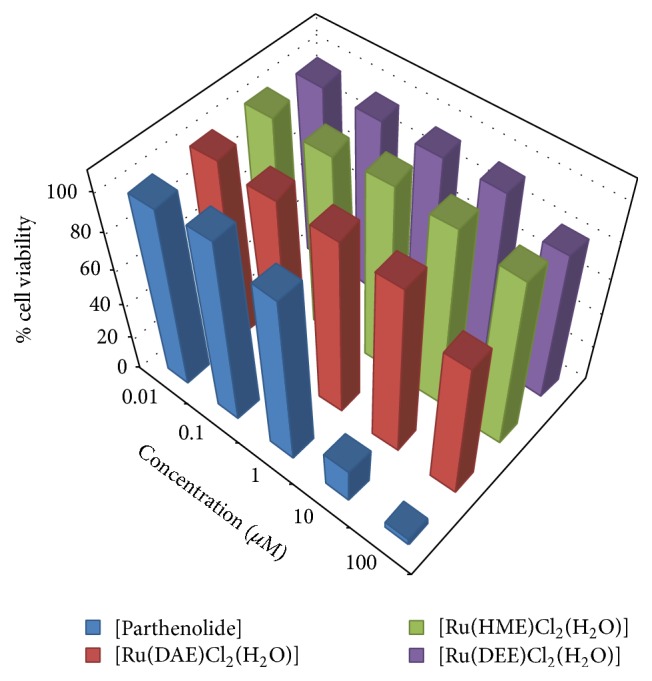
*In vitro* antiproliferative activity of Ru(III) complexes and parthenolide against human renal cancer cell (TK-10).

The ruthenium(III) compounds and standard drug (parthenolide) IC_50_ values are presented in Table 1 and revealed that the test samples showed significant inhibition against the tested cell lines. Figures 2 [Fig fig3]–4 represent the cell viability percentages of ruthenium(III)-Schiff base complexes and parthenolide drug against TK-10, UACC-62, and MCF-7 cell lines, at different concentrations of ruthenium(III) compounds or parthenolide. A high level of antiproliferative potentials against the studied cell lines was exhibited by parthenolide in accordance with earlier reports [45]. The obtained results revealed that treatment of cell lines with different concentrations of Ru(III)-Schiff base complexes efficiently affected cell viability towards MCF-7 cells, as displayed in Figures 2 [Fig fig3]–4 and Table 1. The Ru(III) compounds exhibited low to strong* in vitro* antiproliferative activities against the selected cell lines as compared to the standard drug (parthenolide). [Ru(DAE)Cl_2_(H_2_O)], [Ru(HME)Cl_2_(H_2_O)], and [Ru(DEE)Cl_2_(H_2_O)] induced more efficient cell death with IC_50_ values of 3.57 ± 1.09, 4.88 ± 1.28, and 3.43 ± 1.48 *μ*M, respectively, towards human breast cancer cell (MCF-7) cells than other investigated cell lines, compared with IC_50_ values of 0.44 ± 2.02 *μ*M MCF-7, for the standard cytotoxin drug parthenolide.

The order of activity of the complexes against human melanoma cancer cell (UACC-62) is as follows: [Ru(DEE)Cl_2_(H_2_O)] > [Ru(HME)Cl_2_(H_2_O)] > [Ru(DAE)Cl_2_(H_2_O)]. With respect to previous report by Shier [46], compounds exhibiting IC_50_ activity ranging from 10 to 25 *μ*M are referred to as weak anticancer drugs, while those with IC_50_ action between 5 and 10 *μ*M are moderate, and the compounds possessing activity less than (<) 5.00 *μ*M are considered as strong agents. Thus, the Ru(III) complexes exhibited a weak to strong activity against the investigated cancer cell lines with the following order of activity: MCF-7 > UACC-62 > TK-10. However, [Ru(DAE)Cl_2_(H_2_O)] showed the highest antiproliferative activity with IC_50_ valves of 3.57 ± 1.09, 6.44 ± 0.38, and 9.06 ± 1.18 *μ*M for MCF-7, UACC-62, and TK-10, respectively. The biochemical activity could be due to the methoxy, alkyl, chloride group substituents and bridge spacer: ethylenediamine, which could have played a vital role in antiproliferative potentials of the Ru(III)-N_2_O Schiff base complexes.* In vitro* anticancer activity of the synthesized Ru(III) complexes in this study was compared with Ru complexes reported by other authors and found that [Ru(DAE)Cl_2_(H_2_O)], [Ru(HME)Cl_2_(H_2_O)], and [Ru(DEE)Cl_2_(H_2_O)] complexes exhibited higher antitumor activities. [RuCl(CO)(PPh_3_)L] reported by Raja et al. [47] against human cervical carcinoma cell line, (HeLa) after exposure for 48 h, gave an IC_50_ value in the range of 31.6 *μ*M and [RuCl_2_(AsPh_3_)L] with an IC_50_ value of 37.8 *μ*M [48]. Raju et al. [43] reported ruthenium(III) Schiff base complexes of the type [RuX_2_(PPh_3_)_2_(L)] (where X = Cl or Br; L = monobasic bidentate ligand) complex to have IC_50_ value in the range of 45.2 *μ*M.

### 3.6. Antioxidant Capacity

Different antioxidant techniques and modifications have been put forward to evaluate antioxidants reactivity and functionality in foods and biological systems as a means of checkmating variety of pathological activities such as cellular injury and aging process; these damaging occurrences are caused by free radicals. Hence, two free radicals were used for* in vitro* antioxidants activities of the test samples in this study, namely, 1,1-diphenyl-2-picrylhydrazyl (DPPH) and 2,2′-azino-bis(3-ethylbenzothiazoline-6-sulfonic acid) (ABTS).

#### 3.6.1. DPPH Radical Scavenging Assay

The activity of antioxidants on DPPH radical is believed to be centred on their ability to donate hydrogen [22]. DPPH has been a stable free radical, with the ability to accept hydrogen radical or an electron and then become a stable molecule [49].

**Scheme 2:**
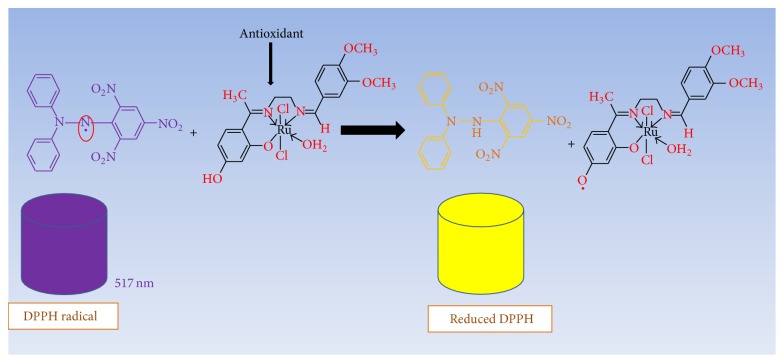
Conversion of DPPH^*∗*^ (purple) to its corresponding hydrazine form (yellow) by the addition of Ru(III) compounds to DPPH^*∗*^ due to proton transfer.

**Figure 5:**
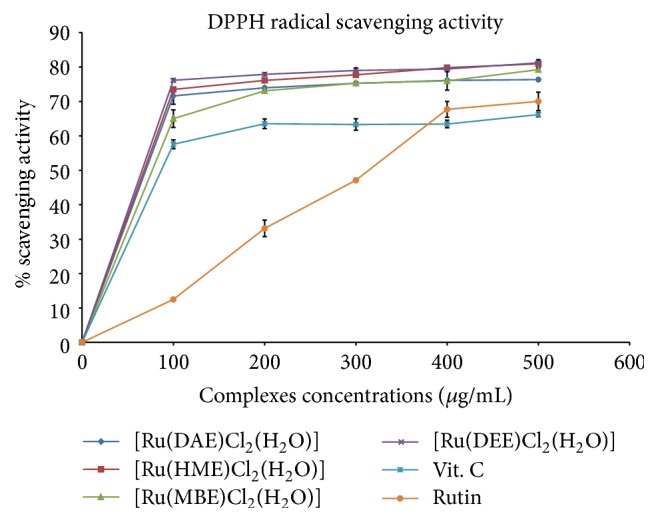
DPPH scavenging potential of Ru(III)-Schiff base complexes.

The mode of rummaging the DPPH radical has extensively been used to appraise antioxidant activities of test samples in a moderately short period of time compared to other procedures [49]. The reduction in the DPPH radical capability is calculated by the decrease in its absorbance at 517 nm prompted by antioxidants [50]. The reduction of DPPH radical intensity in this study is due to the interaction of Ru(III) complexes with radical and as such scavenging the radicals by hydrogen donation (Scheme 2). The DPPH activities by the Ru(III)-N_2_O Schiff base complexes exhibit strong electron donating power when compared to the standards: ascorbic acid and rutin as displayed in Figure 5. The calculated IC_50_ and its corresponding *R* ^2^ (correlation coefficient) values of Ru(III) compounds are listed in Table 2. Compounds [Ru(DAE)Cl_2_(H_2_O)], [Ru(HME)Cl_2_(H_2_O)], [Ru(MBE)Cl_2_(H_2_O)], and [Ru(DEE)Cl_2_(H_2_O)] with an IC_50_ value of 1.60 ± 0.68, 1.54 ± 0.44, 1.63 ± 1.05, and 1.51 ± 0.50 *μ*M, respectively, exhibited higher activity against DPPH than the commercially available Vit. C and rutin (standard); however, [Ru(DEE)Cl_2_(H_2_O)] showed the highest activity of all investigated ruthenium(III) samples with an IC_50_ value of 1.51 ± 0.50 *μ*M.

Scavenging ability of the test samples on the DPPH radical can be ranked in the following order: [Ru(DEE)Cl_2_(H_2_O)] > [Ru(HME)Cl_2_(H_2_O)] > [Ru(DAE)Cl_2_(H_2_O)] > [Ru(MBE)Cl_2_(H_2_O)] > [Vit. C] > [rutin]. The scavenging effect of the DAE, HME, MBE, and DEE ligands is lower as compared to their corresponding Ru(III) complexes, owing to the coordination of the organic molecules to the Ru^3+^ ion. It is further supported by the observed discolouration from purple DPPH radical solution to yellow solution showing scavenging of the DPPH radicals by hydrogen donation (Scheme 2). Hence, these complexes could be effective therapeutic agent's preparation for the treatment of chronic conditions such as cardiovascular, neurodegenerative, and arteriosclerosis diseases [21].

#### 3.6.2. 2,2′-Azino-bis(3-ethylbenzothiazoline-6-sulfonic acid) Radical Scavenging Activity

**Figure 6:**
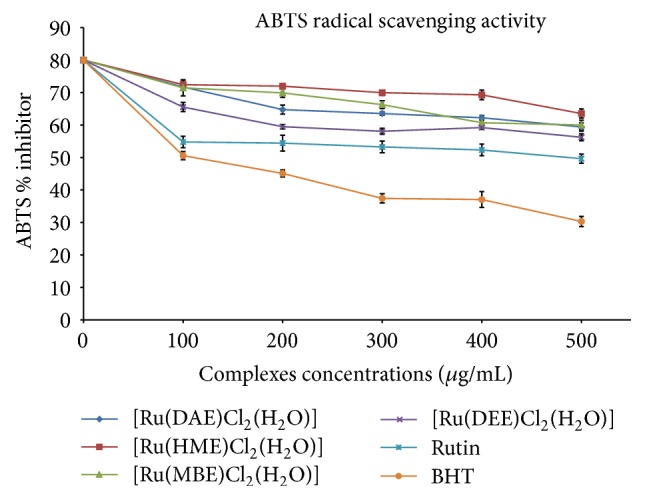
ABTS rummaging activity of Ru(III)-Schiff base complexes.

To further confirm the synthesized Ru(III)-N_2_O Schiff base complexes antiradical potential, we examined the ABTS assay in this study. A well-known protonated radical like 2,2′-azinobis-3-ethylbenzothiazoline-6-sulfonic acid (ABTS) possesses characteristic absorbance maxima at 734 nm and decreases with the scavenging of the proton radicals [51]. The assay measures radical scavenging by electron donation. The outcome of Ru(III)-N_2_O Schiff base complexes alongside the standard drugs on ABTS radical is presented in Table 2. At 734 nm, the absorbance of active ABTS^*∗*^ solution noticeably declined upon the addition of different concentrations of ruthenium(III) samples; the same trend was also observed for the standard drugs: butylated hydroxytoluene (BHT) and rutin hydrate with the percentage inhibition displayed in Figure 6.

The efficacy of the tested samples in quenching ATBS^*∗*^ radicals in the system was observed at 100 *μ*g/mL, the lowest concentration, and Ru(III) complexes exhibited higher ABTS % inhibition than the standards. [Ru(DEE)Cl_2_(H_2_O)] complex exhibited the highest ABTS scavenging activity amongst the studied ruthenium(III) complexes with an IC_50_ value of 3.24 ± 0.93 *μ*M and 0.855 *R* ^2^ (correlation coefficient) as listed in Table 2 while complexes of [Ru(DAE)Cl_2_(H_2_O)], [Ru(HME)Cl_2_(H_2_O)], and [Ru(MBE)Cl_2_(H_2_O)] had an IC_50_ value of 3.30 ± 0.89, 4.27 ± 1.17, and 3.30 ± 1.48 *μ*M, respectively.

The ABTS scavenging activity pattern of the complexes is ranked in the following order: [Ru(HME)Cl_2_(H_2_O)] < [Ru(MBE)Cl_2_(H_2_O)] = [Ru(DAE)Cl_2_(H_2_O)] < [Ru(DEE)Cl_2_(H_2_O)]. With this result, the antiradical studies showed that the synthesised Ru(III)-N_2_O Schiff base complexes may be useful in developing therapeutic agent for averting cell oxidative damage and as radicals chain terminator. This is because various free radicals generated in the system often lead to cancer, cellular injury, aging process, and cardiovascular diseases [21].

## 4. Conclusion

In this study, we present the synthesis of Ru(III) Schiff base complexes formulated as [Ru(LL)Cl_2_(H_2_O)] (LL = DAE, HME, MBE, and DEE). The complexes were characterized using the microanalytical, conductance, electronic, and vibrational spectral analysis. FTIR spectral data showed that the ligand acts as tridentate chelating ligand, coordinating through azomethine nitrogen and phenol oxygen atom. The microanalyses were in conformity with the proposed structures. Conductance measurements showed the complexes to be nonelectrolytes in DMF. Octahedral structures were assigned to these complexes based on the elemental and spectral information.* In vitro* antiproliferative studies of the Ru(III) complexes gave a weak to strong inhibition against the studied cancer cell lines, with the following activity order: MCF-7 > UACC-62 > TK-10. Significantly, further investigation on the compounds free radical scavenging properties revealed that Ru(III)-Schiff base complexes possessed considerable antioxidant activities. The outcome from DPPH and ABTS inhibition studies revealed that the compounds are proficient in donating electron or hydrogen atom and subsequently terminate the chain reactions in a dose-dependent pattern. Scavenging ability of the test samples on the DPPH radicals can be ranked in the following order: [Ru(DEE)Cl_2_(H_2_O)] > [Ru(HME)Cl_2_(H_2_O)] > [Ru(DAE)Cl_2_(H_2_O)] > [Ru(MBE)Cl_2_(H_2_O)]. Thus, Ru(III)-N_2_O Schiff base complexes showed stronger inhibition of DPPH at various concentrations.

## Figures and Tables

**Table 1:** * In vitro* antiproliferative studies of Ru(III)-Schiff base complexes against TK-10, UACC-62, and MCF-7 cell lines.

Compounds	Molecular formula	Anticancer activity IC_50_ (*µ*M) 48 h		
		TK-10	UACC-62	MCF-7
[Ru(DAE)Cl_2_(H_2_O)]	C_18_H_24_N_3_O_4_RuCl_2_	9.06 ± 1.18	6.44 ± 0.38	3.57 ± 1.09
[Ru(HME)Cl_2_(H_2_O)]	C_18_H_23_N_2_O_6_RuCl_2_	41.09 ± 4.44	6.31 ± 1.47	4.88 ± 1.28
[Ru(DEE)Cl_2_(H_2_O)]	C_17_H_20_N_2_O_4_RuCl_3_	13.10 ± 2.81	5.14 ± 1.09	3.43 ± 1.48
Parthenolide^*∗*^	C_15_H_20_O_3_	0.50 ± 1.43	0.89 ± 2.18	0.44 ± 2.02

^*∗*^Standard cytotoxin drug: cell lines were treated with different concentrations of the compounds to achieve 50% inhibition of the culture growth when cultured for 48 h. Value represents mean ± SD of three independent experimentations.

**Table 2:** Radical scavenging abilities (IC_50_ ± SD, *µ*M) of Ru(III)-Schiff base complexes and standard drugs.

Compounds	DPPH radical scavenging activity		ABTS radical scavenging activity	
	IC_50_ (*µ*M)	*R* ^2^	IC_50_ (*µ*M)	*R* ^2^
Ru(DAE)Cl_2_(H_2_O)	1.60 ± 0.68	0.965	3.30 ± 0.89	0.959
Ru(HME)Cl_2_(H_2_O)	1.54 ± 0.44	0.974	4.27 ± 1.17	0.808
Ru(MBE)Cl_2_(H_2_O)	1.63 ± 1.05	0.991	3.30 ± 1.48	0.877
Ru(DEE)Cl_2_(H_2_O)	1.51 ± 0.50	0.963	3.24 ± 0.93	0.855
Rutin^*∗*^	2.52 ± 1.60	0.798	2.83 ± 1.84	0.983
Vit. C^*∗*^	1.92 ± 1.07	0.978	—	—
BHT^*∗*^	—	—	1.64 ± 1.54	0.919

*n* = 3, *X* ± SEM; IC_50_: growth inhibitory concentration; when the inhibition of the tested compounds was 50%, the tested compound concentration was IC_50_. *R* ^2^: correlation coefficient. ^*∗*^Standards.
